# Carbon isotopes of C3 herbs correlate with temperature on removing the influence of precipitation across a temperature transect in the agro‐pastoral ecotone of northern China

**DOI:** 10.1002/ece3.3548

**Published:** 2017-11-03

**Authors:** Xian‐zhao Liu, Yong Zhang, Zhen‐guo Li, Teng Feng, Qing Su, Yan Song

**Affiliations:** ^1^ School of Resource, Environment and Safety Engineering Hunan University of Science and Technology Xiangtan China; ^2^ Institute of Soil Science State Key Laboratory of Soil and Sustainable Agriculture Chinese Academy of Sciences Nanjing China; ^3^ College of Life Science Hunan University of Science and Technology Xiangtan China

**Keywords:** δ^13^C, ANCOVA, herbaceous plants, precipitation influence, soil moisture availability, temperature gradient

## Abstract

Plant δ^13^C–temperature (δ‐T) relation has been established in many systems and is often used as paleotemperature transfer function. However, it is still unclear about the exact contributions of temperature variation to plant ^13^C discrimination because of covariation between temperature and precipitation (aridity), which reduces confidence in reconstruction of paleoclimate. In this study, we measured carbon isotope composition (δ^13^C) of 173 samples of C3 perennial herbs from 22 sites across a temperature gradient along the 400 mm isohyet in the farming‐pastoral zone of North China. The results showed that precipitation obviously affected the correlations of temperatures and foliar δ^13^C. After removing the influence of precipitation by analysis of covariance (ANCOVA), a more strongly positive relationship was obtained between site‐mean foliar δ^13^C and annual mean temperature (AMT), with a regression coefficient of 0.1636‰/°C (*p *=* *.0024). For widespread species, *Artemisia lavandulaefolia* and *Artemisia capillaries*, the slopes (or coefficients) of foliar δ^13^C and AMT were significantly steeper (larger) than those of foliar δ^13^C and AMT where the precipitation influence was not excluded, whereas the δ‐T coefficients of *Polygonum persicaria* and *Leymus chinensis* showed little change across the transect after deducting the precipitation effect. Moreover, the positive relationship between temperature and δ^13^C over the transect could be explained by soil moisture availability related to temperature. Our results may afford new opportunities for investigating the nature of past climate variability.

## 1 INTRODUCTION

Carbon isotope composition (δ^13^C) of terrestrial C3 plants holds important information on internal physiological traits and external environmental changes that affect photosynthetic gas exchange during the time of carbon fixation (Auerswald, Wittmer, Männel, & Schnyder, 2009; Brodribb & Hill, 1998; Zhu, Siegwolf, & Durka, 2010). Owing to its sensitivity to climatic parameters, δ^13^C as a climate proxy is believed to reflect primarily environmental influence and widely used for reconstructions of past climate and paleoenvironment by strong correlations between δ^13^C and parameters reflecting moisture and/or temperature variability (Diefendorf, Mueller, Wing, Koch, & Freeman, 2010; Linares, Delgado‐Huertas, & Carreira, 2011; Werner, Schnyder, Cuntz, Hobson, & Norris, 2012). However, among various univariate δ^13^C–climate relations (Liu, Feng, Ning, & Cao, 2005; Ma, Sun, Liu, & Chen, 2012; Schulze, Turner, Nicolle, & Schumacher, 2006), apparent δ^13^C–temperature relations are most often used as transfer functions, because of the desire to infer paleotemperature.

At present, the influence of precipitation on plants δ^13^C and their relationship have been assessed intensively, with a definite conclusion: δ^13^C of C3 plants generally decreases with increasing precipitation amount (Battipaglia et al., 2014; Kohn, 2010; Maseyk, Hemming, Angert, Leavitt, & Yakir, 2011). However, compared with the unambiguous pattern of the variation of plant δ^13^C with precipitation, uncertain relationships are still existed between δ^13^C in plants and temperature, despite abundant published data. Most studies of C3 plants showed positive correlations between δ^13^C and temperature (McCarroll & Loader, 2004; Schleser, Helle, Lucke, & Vos, 1999; Skrzypek, Kaluzny, & Wojtun, 2007), although few negative and no significant correlations have also been reported. For example, Sheu and Chiu (1995) observed that δ^13^C of C3 plants reduced with increasing temperature, while Gebrekirstos, Worbes, Teketay, Fetene, and Mitlöhner (2009) found no links between δ^13^C and temperature. Most of the above studies were conducted over altitude gradients (Cordell, Goldstein, & Meinzer, 1999; Kogami, Hanba, & Kibe, 2001; Liu, Gao, Su, Zhang, & Song, 2016; Sparks & Ehleringer, 1997; Wang, Zhou, Liu, & Guo, 2010) and hard to effectively separate the influence of temperature from the effects of other environmental factors (such as precipitation and atmospheric CO_2_ concentration) on plant ^13^C discrimination. Accordingly, we know little about how temperature impacts δ^13^C and the contributions of temperature variation to carbon isotopic discrimination in plants. Even though some scholars reported that temperature exerted influence on carbon isotope discrimination in terrestrial plants under controlled conditions of precipitation amount (e.g., annual mean precipitation, AMP), soil moisture, and altitude (Beerling, 1997; Diefendorf et al., 2010; Edwards, Graf, Trimborn, Stichler, & Lipp, 2000; Troughton & Card, 1975), they still could not eliminate the effect of precipitation on carbon isotopes in plants and disentangle the relative contributions of each factor to C isotope discrimination, because of the frequent covariation of temperature and moisture, which leads to poorly constrained or ambiguous assessment of actual isotopic sensitivity to temperature changes. The strong covariation relationships between different environmental variables may also reduce confidence in reconstructions of past climate unless univariate transfer functions of δ^13^C and environmental parameters are known to have been established exactly (Smith, Wing, & Freeman, 2007). Clearly figuring out how temperature and δ^13^C interact and the relative contributions of temperature variation to plant carbon isotopic fractionation is critical to our application of δ^13^C–temperature relations as quantitative transfer functions in paleoclimate reconstructions using carbon isotope records of ancient terrestrial sediment.

In this study, we measured δ^13^C of C3 herbaceous plants across a temperature transect in north China. Our specific objectives were to (1) explore to how plant δ^13^C response to temperature variation; (2) disentangle the relative contributions of temperature factor to carbon isotope fractionation and provide new insight into the possible deconvolution of isotope–climate signals in plants.

## 2 MATERIALS AND METHODS

### 2.1 Study area and transect description

**Figure 1:**
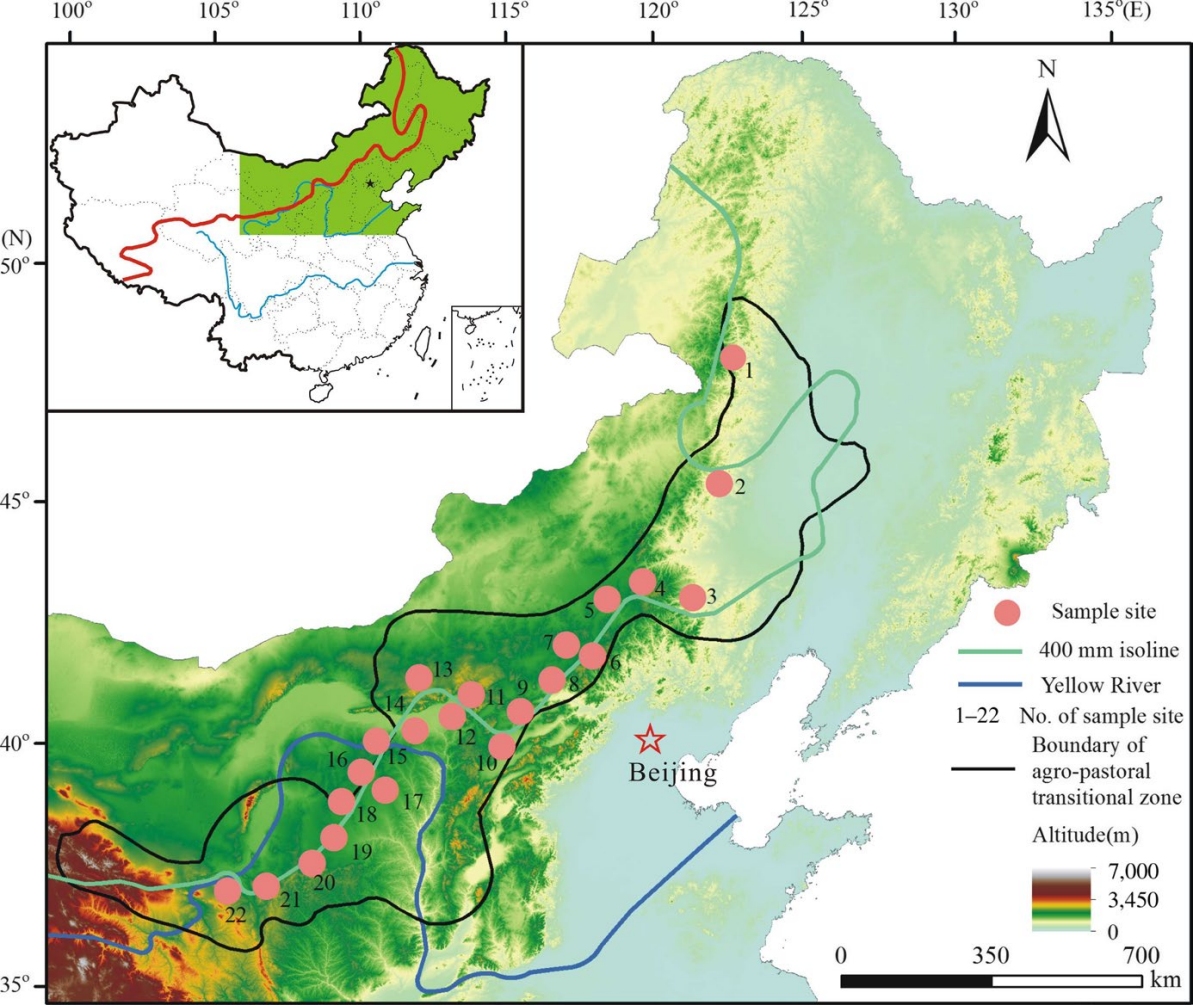
Locations of the sampling sites along the transect in the agro‐pastoral ecotone of north China. Sites are showed as closed pink circles and are numbered as follows: 1, Jinhe; 2, Hailaer; 3, Aershan; 4, Keyouqianqi; 5, Wulanhaote; 6, Bai yanghushuo; 7, Zhaluteqi; 8, Balinzuoqi; 9, Duolun; 10, Baiqi; 11, Fengzhen; 12, Zhungeerqi; 13, Erduosi; 14, Yijinghuoluo; 15, Dongsheng; 16, Youyu; 17, Hequ; 18, Shenmu; 19, Hengshan; 20, Jingbian; 21, Xiji; 22, Yuzhong

The study was conducted in the agro‐pastoral ecotone of north China (APENC, 34°48′—53°26′N, 103°15′—124°37′E), which is located in the southeast of China's Inner Mongolia plateau and north of loess plateau (Figure 1), with a sensitive and fragile ecosystem. To the south of the area is the semihumid north China Plain, which is an intensive agriculture region. To the north is the semiarid temperate steppe along the Inner Mongolian Plateau. The climate of this area is characterized by a distinct transitional nature, with AMP ranging from 300 to 450 mm and AMT varying between 0 and 8°C (Chen, Bai, Lin, Huang, & Han, 2007; Liu, Niu, & Xu, 2013). The spatial variation of temperature follows latitudinal trend. Natural vegetations change from northeast to southwest along with the sequence of the forest–steppe ecotone, typical steppe, and desert steppe. For minimizing the influence of precipitation change on plant C isotope discrimination, we set up a transect with a 15°C difference in annual mean temperature (AMT) along the 400 mm isoline of AMP in the APENC from Jinhe (48.2°N, 121.29°E) in the Inner Mongolia Autonomous Region to Yuzhong (35.92°N, 104.02°E) in Gansu Province. The straight distance between the above two sites is about 1,900 km. Twenty‐two sampling sites were selected along the transect (Figure 1, Table 1). Among these sampling sites, Jinhe (site No. 1) has the lowest AMT of −6.1°C and Shenmu (site No. 18) has the highest AMT of 8.9°C. The obvious temperature gradient provides excellent natural conditions to study the temperature effect on plant δ^13^C. The average AMP of these sites is 394.27 mm, with a standard deviation of ±24.79 mm. The geographic location of each site was recorded using a portable GPS (Garmin, Kansas, USA). Detailed information of the sites is listed in Table 1.

**Table 1:** Information of the sampling sites

No.	Site name	Lon. (E°)	Lat. (N°)	Alt. (m)	AMT (°C)	AMP (mm)	Main soil type	Main vegetation type	Site‐mean δ^13^C (‰)	Sample size (*n*)
1	Jinhe	121.29	48.12	787	−6.1	428.2	Podzolic soil	Temperate meadow steppe	−30.15 ± 0.48	12
2	Hailaer	119.14	47.13	209	−1.0	367.2	Meadow soil	Temperate meadow steppe	−27.49 ± 0.53	8
3	Aershan	119.93	47.20	997	−2.7	379.0	Chernozem	Temperate typical steppe	−27.97 ± 0.37	8
4	Keyouqianqi	121.58	46.05	281	2.1	397.0	Chernozem	Temperate meadow steppe	−27.42 ± 0.65	9
5	Wulanhaote	122.03	46.05	287	4.1	416.7	Drab soil	Temperate meadow steppe	−27.90 ± 0.64	7
6	Bai yanghushuo	121.27	45.04	280	7.3	375.2	Kastanozem	Temperate typical steppe	−25.21 ± 0.57	5
7	Zhaluteqi	120.90	44.57	265	2.8	387.6	Kastanozem	Temperate meadow steppe	−28.57 ± 0.47	7
8	Balinzuoqi	119.06	43.98	486	5.3	390.0	Kastanozem	Temperate typical steppe	−26.83 ± 0.62	9
9	Duolun	116.47	42.18	1,245	2.4	407.0	Kastanozem	Temperate meadow steppe	−28.29 ± 0.71	10
10	Baiqi	115.12	42.24	1,405	2.0	363.0	Kastanozem	Temperate typical steppe	−26.33 ± 0.21	4
11	Fengzhen	113.45	40.26	1,195	4.7	413.0	Heilu soil	Temperate meadow steppe	−28.08 ± 0.51	8
12	Zhungeerqi	110.26	39.03	1,249	7.5	400.0	Loessial soil	Temperate meadow steppe	−25.86 ± 0.45	4
13	Erduosi	110.47	39.35	1,108	6.4	335.0	Loessial soil	Temperate typical steppe	−26.59 ± 0.52	8
14	Yijinghuoluo	110.05	39.17	1,276	6.2	365.0	Loessial soil	Temperate typical steppe	−26.47 ± 0.66	6
15	Dongsheng	109.98	39.03	1,461	5.4	400.0	Loessial soil	Temperate typical steppe	−26.61 ± 0.73	11
16	Youyu	112.27	40.00	1,358	8.2	442.8	Kastanozem	Temperate meadow steppe	−27.92 ± 0.56	8
17	Hequ	111.15	39.38	875	8.8	426.0	Loessial soil	Temperate meadow steppe	−27.46 ± 0.53	6
18	Shenmu	109.54	38.24	1,226	8.9	393.0	Loessial soil	Temperate meadow steppe	−27.19 ± 0.38	6
19	Hengshan	109.17	37.28	1,019	8.5	397.0	Loessial soil	Temperate typical steppe	−26.69 ± 0.46	8
20	Jingbian	108.50	37.36	1,333	7.8	395.0	Loessial soil	Temperate typical steppe	−27.65 ± 0.60	10
21	Xiji	105.44	37.57	1,931	5.3	400.0	Loessial soil	Temperate meadow steppe	−26.25 ± 0.77	10
22	Yuzhong	104.02	35.92	1,896	6.6	403.0	Heilu soil	Temperate meadow steppe	−26.51 ± 0.71	9

Lon, Lat, Alt, AMT, and AMP are the abbreviations of longitude, latitude, altitude, annual mean temperature, and annual mean precipitation, respectively. AMT and AMP represent the average values of more than 30 years. Dominant soil and vegetation types were from “1:1,000,000 Soil Map of China” (2007) and “1:1,000,000 Vegetation Atlas of China” (2001), respectively.

### 2.2 Plant sampling

Plants were sampled along the 400 mm isoline of AMP (Figure 1) in the summer of 2008 from July 28 to August 30, when all plants were mature. The species collected could be divided into two groups. The first group (noneurytopic species) comprised those with high abundance in the community but limited distribution across the transect (only, a few species were not sampled because they occupied one site or community); the second group that occurred widely throughout the transect, included the four representative species *Artemisia lavandulaefolia*,* Artemisia capillaries*,* Polygonum persicaria,* and *Leymus chinensis*. In order to exclude the influences of leaf age (lifespan) on C isotope discrimination, here, we only chose the perennial C3 herbs for our survey. At each site, five to seven individuals of each species, which were restricted to sites far from human habitats, were identified and the same number of mature sun‐exposed leaves collected from each individual. Leaves that were incomplete or too large or small were excluded for the sake of homogeneity of pooled samples. The leaves from each species were pooled to form one sample at each site. A total of 173 samples were collected, including 101 samples of 40 dominant or codominant species and 72 samples of four eurytopic plants.

### 2.3 Measurement of plant δ^13^C values

Plant samples were taken back to the laboratory, washed in distilled water, and dried at 70°C for 48 hr. Oven‐dried samples were finely ground and sieved with 80‐mesh sieve. The weighed pulverized sample (3—5 mg) was put into a sealed vacuum tube and combusted at a temperature of 1,020°C for producing CO_2_. Plant isotope ratios were determined by a Delta‐^plus^XP mass spectrometer (Thermo Scientific, Bremen, Germany) coupled with an elemental analyzer (FlashEA1112, CE Instruments, Wigan, UK) in continuous flow model. Carbon isotopic value is expressed as the standard notation relative to the Vienna Pee Dee Belemnite standard using the following equation: δ^13^C = (*R*_sample_/*R*_standard_−1) × 1,000 (‰), where *R*_sample_ and *R*_standard_ are the ^13^C/^12^C ratios of the sample and the standard, respectively. The overall precision of the delta values was about 0.2‰, as determined by repetitive measurements of standard material.

### 2.4 Meteorological data

Annual mean temperature (AMT) and annual mean precipitation (AMP) at each site were obtained from the nearest available meteorological station. AMT and AMP represented the average values of more than 30 years (from 1978 to 2008).

### 2.5 Data analysis

In this study, although we tried to collect plant samples along the 400 mm isoline, not all of these sites experienced rainfall of 400 mm (Table 1). Hence, carbon isotopic data of C3 herbs should contain trends and variability related to the precipitation. To eliminate the impact of precipitation on C3 herbs δ13C, analysis of covariance (ANCOVA) was conducted by the SAS 9.2 software package (SAS Institute Inc. NC, USA), with δ13C as a dependent variable, temperature as an independent factor, and precipitation as a covariate (Correia et al., 2008; Ogaya & Penñuelas, 2008). There was a simple two‐step process. First, we performed linear regression of δ13C against precipitation to test whether the variation of δ13C was influenced by precipitation. If there was a significant regression relationship between the variables being studied, we can obtain a covariance parameter (a correction coefficient) of annual precipitation‐δ13C to correct for the influence of precipitation. Second, we calculated the δ^13^C values of all samples employing the precipitation correction equation $\delta_{\mathrm{CB}}^{13}=\delta_{\mathrm{CA}}^{13}+\beta \times \left(\text{AMP}-{\text{AMP}}_{A}\right)$, where $\delta_{\mathrm{CA}}^{13}$ denotes the raw δ^13^C value; $\delta_{\mathrm{CB}}^{13}$ is the δ^13^C value after the precipitation correction; β is the correction coefficient, namely covariance parameter; AMP is the annual mean precipitation at each site; AMPA is the average AMP of all sites (More detailed description of ANCOVA can be found in Liu, Zhang, and Li, 2009.). The corrected δ^13^C values across samples within each site were averaged to obtain a site‐averaged δ^13^C series (Table 1). The site‐mean values of site series were used to test their correlation with annual mean temperature (AMT) by linear regression. Differences among regression slopes were determined using Standardised Major Axis Estimation & Testing Routines (SMATR3.4‐3), a freely available program. One‐way analysis of variance was used to test the differences in δ^13^C values between removing precipitation influence and without eliminating precipitation effect at each site by the significant level at *p *<* *.05.

## 3 RESULTS

### 3.1 Variation in foliar δ^13^C of C3 herbs along the transect

**Figure 2:**
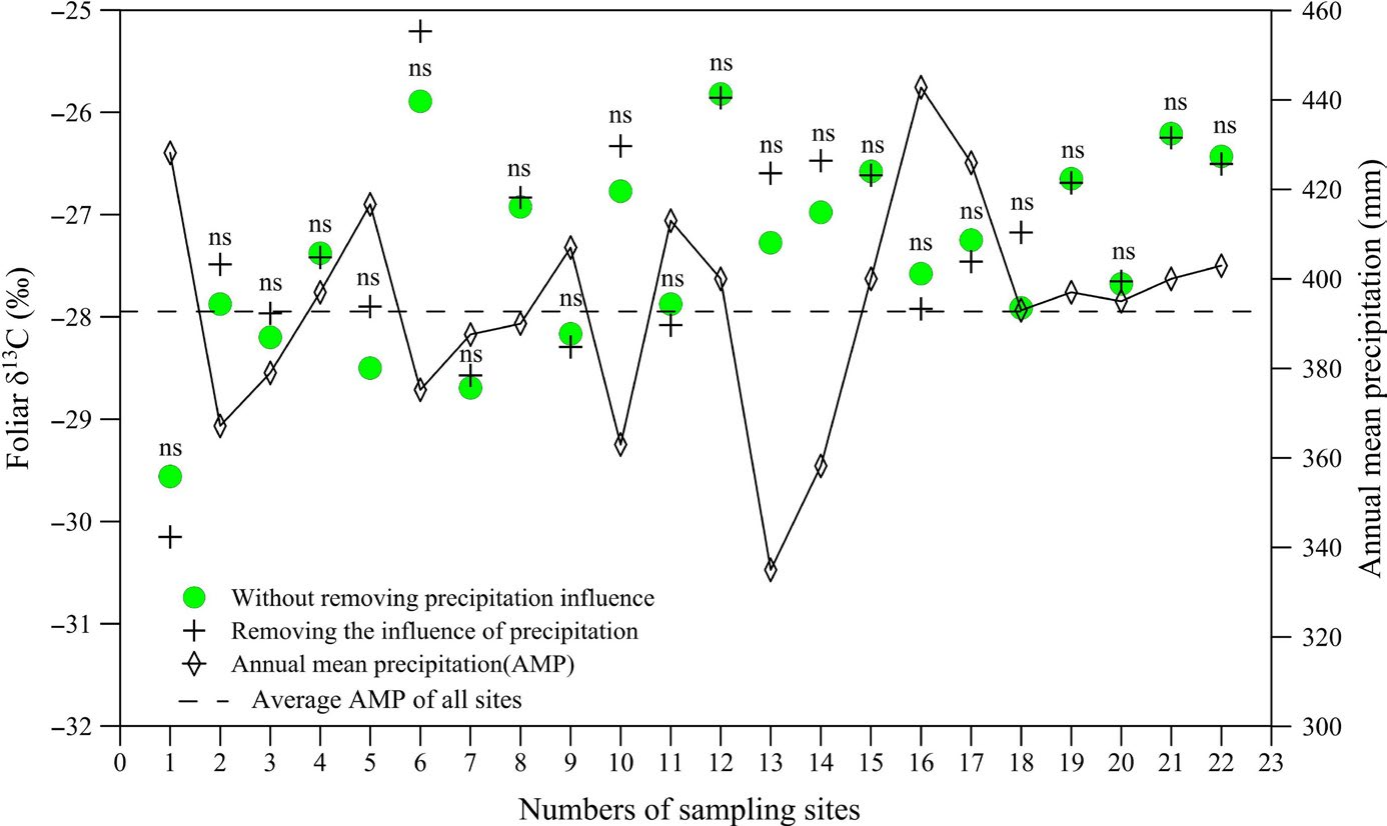
Variation of foliar δ^13^C along the transect in the agro‐pastoral ecotone of north China. The ns means not significant at the α = 0.05 level. Each point is the mean foliar δ^13^C of all samples within each site

The δ^13^C values of C3 herbs in the present study ranged from −24.98‰ to −31.19‰ with a mean value of −27.48‰ (*n* = 173, *SD* = 1.34). After removing the potential precipitation effect, the range of δ^13^C values, varying from −24.69‰ to −31.41‰, basically fell within the range (−22‰ to −34‰) of values observed in terrestrial C3 plants (Vogel, 1993). However, the mean value (−27.43‰) obtained from the investigated species in our study was distinct lower than that of C3 plants from a global scale (−27.0‰). This inconsistency in mean δ^13^C values could be attributed to the presence or absence of woody plants, because the δ^13^C value of woody species is about 2‰ higher than that of C3 herbs (Kloeppel & Gower, 1998). Within each site, when the annual mean precipitation is less than the average annual mean precipitation of all sampling sites, the site‐averaged δ^13^C value removing the influence of precipitation was higher (more positive) than one without removing the influence of precipitation and vice versa, but no significant difference existed between the two (Figure 2). It indicated that slight precipitation difference did not significantly affect δ^13^C of C3 herbs, because our plant samples were collected along the 400 mm isoline of annual mean precipitation (AMP) and the average AMP of these sites is 394.3 mm, with a standard deviation of ±24.79 mm.

### 3.2 Responses of foliar δ^13^C to temperature

**Figure 3:**
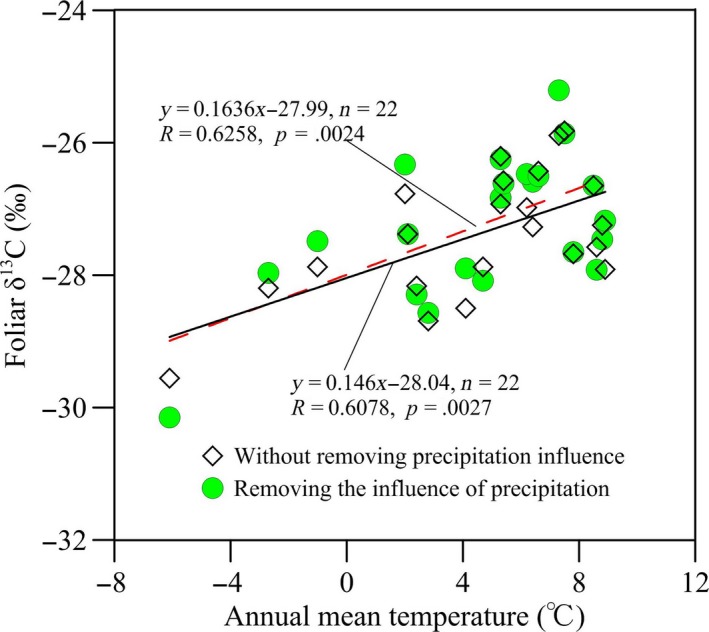
Response of foliar δ^13^C for C3 herbs to annual mean temperature (AMT) across the transect. The red dotted line represents the fitting line between foliar δ^13^C and the temperature where the precipitation influence was excluded, and the black solid line represents the fitting one where the precipitation influence was not excluded. Each point is the mean foliar δ^13^C of all samples within each site

As shown in Figure 3, the annual mean temperature (AMT) had a significant positive relationship with δ^13^C of collected C3 herbs in this study, and when ANCOVA was used to statistically remove the influence of precipitation, this relationship became stronger than that for the raw δ^13^C without separating the potential precipitation effect. The slope of the regression between δ^13^C removing the effect of precipitation (corrected δ^13^C) and AMT in the transect was 0.1636‰/°C, which was much larger than the one between raw δ^13^C and AMT (slope *= *0.146‰/°C). However, both the two slopes and the *P*‐values of slope tests did not differ significantly (*p = *.084 and .137, respectively; Figure 3). The correlation coefficients of corrected δ^13^C and raw δ^13^C for AMT were also not significantly different (*p = *.103).

**Figure 4:**
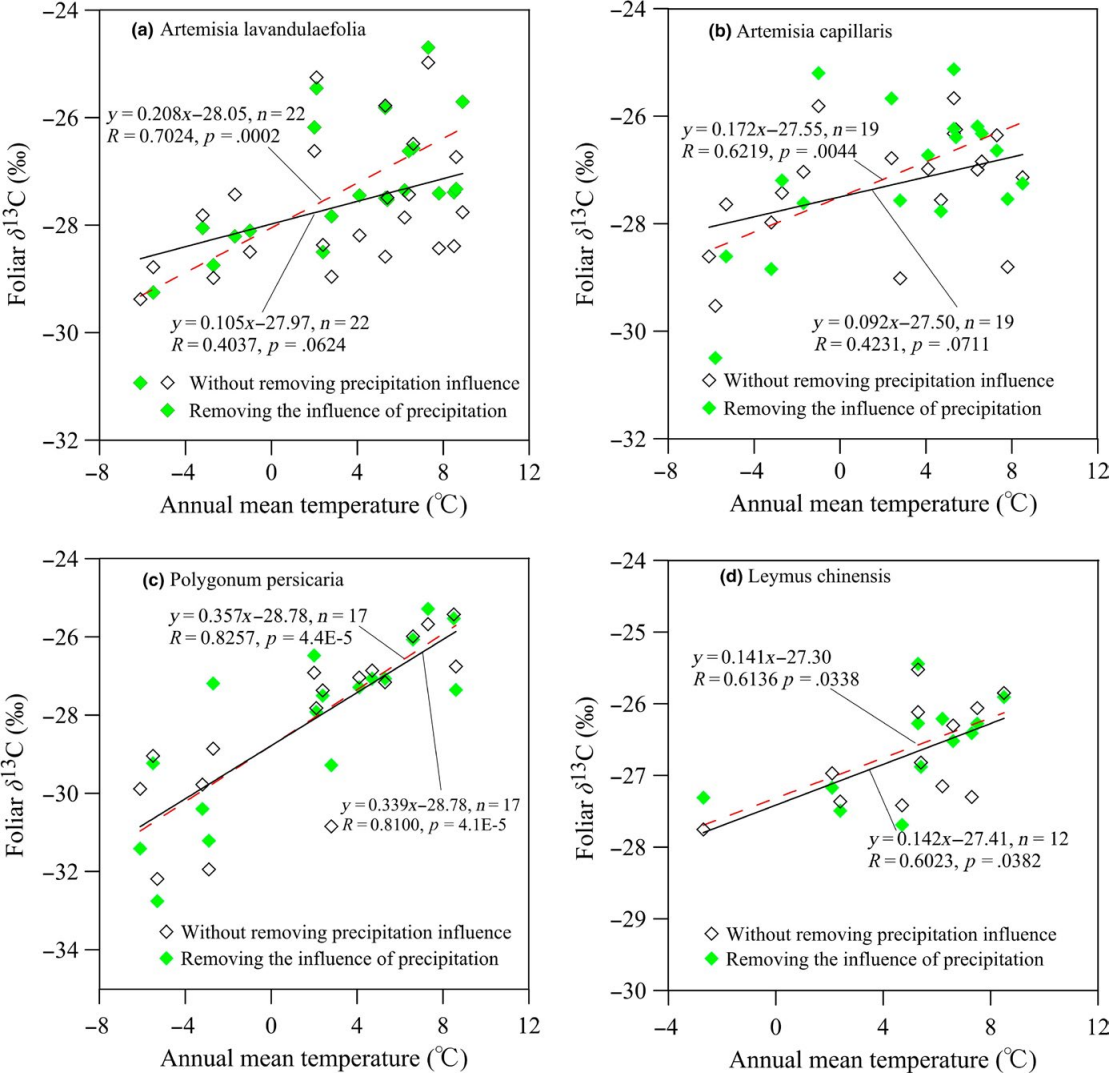
Response patterns of foliar δ^13^C for eurytopic C3 herbs to annual mean temperature (AMT) along the transect. The red dotted line represents the fitting line between foliar δ^13^C and the temperature where the precipitation influence was excluded, and the black solid line represents the fitting one where the precipitation influence was not excluded. Each point is the mean foliar δ^13^C of all samples within each site

Similar positive‐response relationships were also found between the δ^13^C values and AMT for the four C3 eurytopicity species. We detected that all eurytopic species displayed increasing δ^13^C with rising temperature (Figure 4). After removing the potential precipitation effect, the slopes of the regression between δ^13^C and AMP were extremely significant for *Artemisia lavandulaefolia* (*p *=* *.0002; Figure 4a) and *Artemisia capillaries* (*p *=* *.0044; Figure 4b), and the correlation coefficients of corrected δ^13^C for temperature were significantly higher when compared with ones of raw δ^13^C for temperature (*p *=* *.024 and .037, respectively; Figure 4a,b). However, it was worth noting that correlation coefficient and regression slope between corrected δ^13^C and AMT showed little changes for *Polygonum persicaria* and *Leymus chinensis*, although there were lower *p*‐values of the slope tests for them (Figure 4c,d). This suggested that the four species have different sensitivities to temperature and precipitation.

## 4 DISCUSSION

Precipitation affected the relationships between δ^13^C and temperatures. We detected that overall foliar δ^13^C strongly positively correlated with AMT along the transect regardless of whether it was with or without statistically removing the influence of precipitation, which showed that C3 herbs have significant responses to temperature variation, and corroborated the general pattern previously reported by numerous researchers (Liu, Zhao, Gasaw, Gao, & Qin, 2007; Loader & Hemming, 2001; McCarroll & Loader, 2004; Schleser et al., 1999; Skrzypek et al., 2007). This result also highlights the potential of using δ^13^C of plant matter as a temperature proxy for paleoclimate reconstruction. Nevertheless, the differences in regression slopes and correlation coefficients of the corrected δ^13^C (removing precipitation effect) and the raw δ^13^C (without removing precipitation influence) with AMT were not statistically significant, although there were significant correlations between the δ^13^C values and the temperatures (Figure 3). An explanation for this could be that the AMP of each site was very close to the average AMP of all sites (Figure 2), and when covariance analysis was used to correct for the impact of precipitation, the corrected δ^13^C was calculated based on the difference between annual precipitation amount of each site and average AMP of all sites. Interestingly, however, after deducting the effect of precipitation on C3 herbs δ^13^C, the slope of foliar δ^13^C and AMT along the transect (0.1636‰/°C) was larger (steeper) when compared to the “corrected” slope of 0.104‰/°C for C3 species, as reported by Wang, Li, Liu, and Li (2013). The distinctly greater value of our slope might be attributed to the selection of the samples and method of excluding the effect of precipitation on plants δ^13^C in our study. The results of Wang et al. (2013) may be affected by noncongeneric samples (including shrub, grass, and tree) because significant δ^13^C differences exist among different life‐form plants and among different lifespan herbs, and no isotopic offset was conducted for these data before regression analysis. Furthermore, the coefficient of AMP‐δ^13^C adopted for precipitation correction could not be the most effective. Because the coefficient of AMP‐δ^13^C was derived mainly from the middle of Loess Plateau with annual precipitation of 480–600 mm, and the climatic conditions of most sampling sites are different from those of the study area in Wang et al. (2013). Additionally, our findings were also inconsistent with the observations of Gebrekirstos et al. (2009) and Diefendorf et al. (2010), which suggested that temperature exerted negative or only minimal effects on plant δ^13^C. The inconsistency might be attributed to either the limited data (including imperfect δ^13^C data sources) or number of species.

For four eurytopic species, they all displayed increasing δ^13^C with rising temperature, but the regression slope was obviously different from each other (Figure 4). The slope was largest for *Polygonum persicaria* (*p *=* *4.1E−5) and smallest for *Artemisia capillaries* (*p *=* *.0711), suggesting that different species might respond differently to temperature. When ANCOVA was used to remove the influence of precipitation, plant δ^13^C was extremely positively correlated with AMT for *Artemisia lavandulaefolia* and *Artemisia capillaries*, and their slopes of foliar δ^13^C and AMT was significantly steeper than those without excluding the effect of precipitation (Figure 4a,b). From this, we infer that precipitation could strongly drive δ^13^C variation of the two above‐mentioned species and must be accounted for when analyzing the relationship between temperature and δ^13^C. However, correlations of δ^13^C and temperature for *Polygonum persicaria* and *Leymus chinensis* changed little after the precipitation correction by ANCOVA, although high correlations were always present (Figure 4c,d). This indicated that foliar δ^13^C of the two common C3 species was insensitive to changes in precipitation. One plausible explanation for this was that, in addition to the plasticity pattern that has arisen, there was a genetically determined pattern of foliar δ^13^C (Funk & Vitousek, 2007). Some reports showed that δ^13^C in plant tissues was controlled by genes located in different chromosomes (Raddad & Lukkanen, 2006). The transect in the present study is a typical transition zone with a large temperature gradient and has an annual average rainfall of ca. 400 mm. Over the thousands of kilometers long transects, plants encounter a variety of microclimates differing in temperature, precipitation, and soil moisture, each of which may influence δ^13^C. A species can persist in a heterogeneous environment either by means of phenotypic plasticity or genetic difference present among individuals (Saurer, Siegwolf, & Schweingruber, 2004). Considering that among the four eurytopic herbs, *Leymus chinensis* represents only little genetic variation across the transect (Liu et al., 2013), and it was reasonable to attribute the observed temperature trend in δ^13^C mainly to individual phenotypic plasticity. That is because plastic responses, expressed as morph‐physiological adaptations, are those that influence physiological functions, such as photosynthesis and stomatal conductance and, hence, δ^13^C. Therefore, a plastic response would further increase the foliar δ^13^C with increasing temperature or in drought‐prone habitats.

Numerous investigations have demonstrated that environmental factors, particularly growth‐limiting factors, are generally responsible for variation in plant δ^13^C. Precipitation is considered to be an important factor determining carbon isotope fractionation. Water deficit usually causes a reduced Ci/Ca ratio and decreasing plant ^13^C discrimination, leading to increases in δ^13^C values (Battipaglia et al., 2014; Ripullone, Guerrieri, Saurer, Siegwolf, & Jäggi, 2009). Such a negative correlation between δ^13^C of C3 plants and AMP has been reported widely (Devitt, Smith, & Neuman, 1997; Diefendorf et al., 2010; Kohn, 2010; Liu et al., 2016). In our study, however, although precipitation can drive δ^13^C variation, we argued that the influences of precipitation were unlikely to result in significant differences in δ^13^C among sites. One reason for this was that all the samples in the present study were roughly collected along the 400 mm isoline in north China, and the difference in rainfall amount between these sites was very small. Another reason could be that precipitation was not an efficient indicator of water availability to plants in this case because soil water availability is also affected by many environment factors other than precipitation, such as temperature, wind speed, and soil types.

However, we considered temperature as a control factor that affected the carbon isotope fractionation of plants along the transect. According to the theory proposed by Morecroft and Woodward (1996), the effect of temperature on ^13^C discrimination was carried out by affecting enzyme activity as well as stomatal conductance, assimilation rate, and the ratio of Ci/Ca. Lowing temperature generally leads to a reduction in enzyme activity and a lower leaf‐to‐air vapor pressure deficit, which also causes stomata to open, resulting in an increase in Ci/Ca ratio and decreasing δ^13^C values (Devitt et al., 1997). In this study, we proposed that temperature‐related soil moisture availability could explain the positive correlation between plant δ^13^C and temperature. This belief was based on two considerations. First, Temperature is a critical factor determining evaporation, which is closely related to soil moisture. Although the annual rainfall amount was almost the same for each sampling site, the temperatures varied greatly across the transect, and this would cause a big difference in soil water availability among sites. When precipitation supply was insufficient, soil moisture availability would reduce. Plants may more often be reducing stomatal conductance to prevent excess water loss, which leads to a decreasing Ci/Ca ratio and thus an increasing δ13C. Second, relative to precipitation, soil moisture is more directly correlated with plant δ13C. As soil moisture at each site was measured only once, we lacked the persuasive data to analyze the relationship between carbon isotope ratios and soil moisture.

In addition to temperature and precipitation (soil moisture), many abiotic factors, such as altitude, longitude, and latitude, can affect plant δ^13^C. However, when estimating the correlation of plant δ^13^C and temperature, we believe that these effects may be limited and even can be neglected. The reason for this is that the variations of altitude, longitude, and latitude tend to cause changes in temperature and/or precipitation; therefore, their effects on plant δ^13^C should be embodied in the effects of temperature and precipitation. Other environmental factors such as solar radiation and air pressure will also vary with altitude (longitude and latitude), but it is generally considered that the altitudinal trend of plant δ^13^C can be attributed mainly to the impacts of temperature and/or precipitation rather than to changes in atmospheric pressure and solar radiation (Sparks & Ehleringer, 1997; Zhu et al., 2010). The differences in sunshine hours associated with cloud cover among sites might generate changes in plant δ^13^C. However, here, we did not segregate the effects of sunshine hours—from temperature‐related plant δ^13^C because all sampling sites are located in the area where the terrain is mostly flat and free of obstructions such as trees and buildings. We were confident that sunshine‐related differences in δ^13^C among sites could be ignored.

## 5 CONCLUSIONS

This study obtained an unbiased coefficient of AMT‐δ^13^C through measuring δ^13^C of a large number of congeneric samples (all the C3 perennial herbs) from the farming‐pastoral ecotone in north China. After removing the effects of precipitation from temperature influences, a more strongly positive correlation appeared between site‐averaged δ^13^C and AMT, with a slope (coefficient) of 0.1636‰/°C. For widespread species, when ANCOVA was used to deduct the precipitation effect, δ^13^C was extremely positively correlated with temperature for *Artemisia lavandulaefolia* and *Artemisia capillaries* except for *Polygonum persicaria* and *Leymus chinensis*, whose correlations between δ^13^C and temperature changed little. In water‐limited conditions, we believed that the positive relationship between temperature and δ^13^C over the whole transect could be explained by soil moisture availability related to temperature, although temperature is the main factor affecting δ^13^C of C3 herbs. The temperature‐related change in δ^13^C of individual species was a result of environmentally induced responses in ecophysiological process. In conclusion, the influence of precipitation should be considered in research on the relation of temperature and plant δ^13^C. Future research aiming to exhaustively explain the observed temperature trends in δ^13^C should involve direct measurement of phenotypic plasticity and genetic variation.

## CONFLICT OF INTERESTS

All authors declare that we do not have any competing financial interests.

## AUTHOR CONTRIBUTIONS

XZL and YZ designed the experiment; XZL wrote the manuscript; ZGL and TF contributed to map‐making; QS and YS provided editorial advice.
